# Supplementary material for: Empirical methods for controlling false positives and estimating confidence in ChIP-Seq peaks
Source: BMC Bioinformatics. 2008 Dec 5;9:523. doi: 10.1186/1471-2105-9-523 (PMC2628906; doi:10.1186/1471-2105-9-523)
Supplement: Additional File 1 — A variety of html documents from the USeq web site detailing the available applications, their best usage, output file type descriptions, command line menus, etc. [file 1471-2105-9-523-S1.zip › USeqUserGuides/JavaDoc.zip!/JavaDoc/index-files/index-19.html]

T-Index


|  |  |  |
| --- | --- | --- |
| **Overview** \| Package \| Class \| Use \| **Tree** \| **Deprecated** \| **Index** \| **Help** | |  |
| **PREV LETTER**   **NEXT LETTER** | **FRAMES**    **NO FRAMES**     **All Classes** |  |


A B C D E F G H I J L M N O P Q R S T U V W X Z 

---


## **T**

**T2** - Class in trans.main: Runs entire TiMAT2 suite based on a parameter file. **T2(String[])** - Constructor for class trans.main.T2: **T2Parameter** - Class in trans.main: Container with info related to runing multiple T2 programs. **T2Parameter(File)** - Constructor for class trans.main.T2Parameter: **TableHeading** - Class in bioroot: **TableHeading(String, int)** - Constructor for class bioroot.TableHeading: **tableLastModified(String)** - Method in class bioroot.DBUtil: **Tag2Point** - Class in edu.utah.seq.parsers: Splits a tag file (chr start stop ... **Tag2Point(String[])** - Constructor for class edu.utah.seq.parsers.Tag2Point: **TARGET\_MEDIAN** - Static variable in class trans.bkgrnd.MapControlIntensities: **TASSignalChromFileSplitter** - Class in util.apps: **TASSignalChromFileSplitter()** - Constructor for class util.apps.TASSignalChromFileSplitter: **test(float[], float[])** - Method in class trans.main.WilcoxonRankSumTest: Returns a -10LogBase10(pValue) if n1 and n2 >9. **test(float[][], float[][], int, int)** - Method in class trans.main.WilcoxonRankSumTest: Returns a -10LogBase10(pValue) if n1 and n2 >9 **test()** - Method in class trans.main.WilcoxonSignedRankTest: **testCelFiles()** - Method in class trans.qc.CelFileQualityControl: Main app controler. **TestInBioTools** - Class in cgar: **TestInBioTools()** - Constructor for class cgar.TestInBioTools: **testOverlap(Interval[], Interval[], File, File)** - Method in class trans.main.OverlapCounter: Returns the average % intersection. **TestScanChip** - Class in trans.main: Scores windows of intensity values using several tests. **TestScanChip(String[])** - Constructor for class trans.main.TestScanChip: **TextFrame** - Class in util.gen: Creates a free standing plain editable text window. **TextFrame(int, int, int, int, String)** - Constructor for class util.gen.TextFrame: **TextParser** - Class in util.apps: For parsing a text file into a tab delimited file, working template. **TextParser()** - Constructor for class util.apps.TextParser: **TextPValOrSig2Gr** - Class in trans.misc: Converts a bed file to multiple gr files. **TextPValOrSig2Gr()** - Constructor for class trans.misc.TextPValOrSig2Gr: **TextWindow** - Class in gata.main: **TextWindow(String, int, int, String)** - Constructor for class gata.main.TextWindow: **threadedResults** - Variable in class util.amazon.S3Manager: **thresholdWindows(Window[], double, int)** - Method in class trans.main.FDRWindowConverter: **throwError(JFrame, String)** - Static method in class util.gen.Swing: **throwWarning(JFrame, JTextField, String)** - Static method in class gata.main.GATAUtil: **throwWarning(JFrame, JTextField, String)** - Static method in class util.gen.Swing: **toHTMLString()** - Method in class gata.aligner.AlignParams: **toLowerCase(String[])** - Method in class util.gen.Misc: Lower cases a String[] **ToolsFrame** - Class in gata.plotter: **ToolsFrame(int, int, GATAParams)** - Constructor for class gata.plotter.ToolsFrame: **toString()** - Method in class bioroot.Feature: **toString()** - Method in class bioroot.LabGroup: **toString()** - Method in class bioroot.PatternMatch: **toString()** - Method in class bioroot.SearchHit: **toString()** - Method in class bioroot.TableHeading: **toString()** - Method in class edu.utah.seq.data.Info: **toString()** - Method in class edu.utah.seq.data.Point: **toString()** - Method in class edu.utah.seq.methylation454.Amplicon: **toString()** - Method in class edu.utah.seq.methylation454.BaseRead: **toString()** - Method in class edu.utah.seq.methylation454.CpG: **toString()** - Method in class edu.utah.seq.methylation454.DataLine: **toString()** - Method in class edu.utah.seq.methylation454.Sample: **toString()** - Method in class edu.utah.seq.parsers.BarParser: **toString()** - Method in class expr.CorrelationWindow: Genes NumGenes Chromosome Start Stop CorrelationScore -10Log10(pvalue) **toString()** - Method in class expr.ExpressedGene: **toString()** - Method in class expr.MiRNA: **toString()** - Method in class expr.MiRNATarget: **toString()** - Method in class gata.aligner.AlignerPreferences: **toString()** - Method in class gata.geneGlyphs.GeneRepGlyph: **toString()** - Method in class gata.geneGlyphs.GenericGlyph: **toString()** - Method in class meme.MemeMotif: **toString()** - Method in class meme.MemeParser: **toString()** - Method in class meme.MemeResults: **toString()** - Method in class meme.MotifHit: **toString()** - Method in class trans.anno.BindingRegion: **toString()** - Method in class trans.anno.Region: **toString()** - Method in class trans.bkgrnd.BackGroundOligo: **toString()** - Method in class trans.cel.ControlStats: **toString()** - Method in class trans.cel.MultiSetQuantile: **toString()** - Method in class trans.graphics.GenomicRegion: Returns tab delimited rank+1, chrom, st, stp, scr, notes. **toString(Oligo[], int)** - Method in class trans.main.BindingPeak: Score, Left, Peak, Right **toString()** - Method in class trans.main.Interval: **toString()** - Method in class trans.main.Oligo: **toString(int, int, String)** - Method in class trans.main.Oligo: **toString()** - Method in class trans.main.WilcoxonSamplePair: **toString()** - Method in class trans.main.WilcoxonSignedRankTest: **toString()** - Method in class trans.qc.CelFileStats: **toString()** - Method in class trans.qc.StatFlag: **toString()** - Method in class trans.roc.Bin: **toString()** - Method in class trans.roc.Gr: **toString()** - Method in class trans.roc.Positive: **toString()** - Method in class trans.roc.Sgr: **toString()** - Method in class trans.tpmap.TPMapFeature: **toString()** - Method in class util.apps.gump.Option: **toString()** - Method in class util.apps.HouseBean: **toString()** - Method in class util.bio.annotation.Coordinate: **toString()** - Method in class util.bio.annotation.ExonIntron: **toString()** - Method in class util.bio.annotation.GeneGroup: **toString()** - Method in class util.bio.annotation.GeneRep: **toString()** - Method in class util.bio.annotation.StartStop: **toString()** - Method in class util.bio.annotation.Transcript: **toString()** - Method in class util.bio.annotation.TransGroup: **toString()** - Method in class util.bio.annotation.Translation: **toString()** - Method in class util.bio.parsers.BaseCount: **toString()** - Method in class util.bio.parsers.gff.GadFlyGffExtractor: **toString()** - Method in class util.bio.parsers.gff.Gff3Feature: Generates a GFF3 line. **toString()** - Method in class util.bio.parsers.gff.GffFeature: **toString()** - Method in class util.bio.parsers.UCSCGeneLine: **toString()** - Method in class util.bio.wrappers.BlastResult: **toString()** - Method in class util.gen.Bin: **toStringALInts(ArrayList)** - Static method in class util.bio.annotation.Translation: **toStringBarGraph(int[])** - Method in class util.bio.xamer.Xamer: Run pairwise comparisons between percentHitsPerRound if any round is less than the others return true public boolean isEnriched(){ int tester =0; int len = percentHitsPerRound.length; for (int i=0; i**toStringNoAttributes()** - Method in class util.bio.parsers.gff.Gff3Feature: Generates a GFF3 line. **toStringPercent(int[])** - Method in class util.bio.xamer.Xamer: **toStringSimple()** - Method in class trans.roc.Positive: **totalBP(StartStop[])** - Static method in class util.bio.annotation.StartStop: Returns the total number of bases, assumes interbase coordinates. **totalLength(Object[][])** - Static method in class util.gen.Misc: Counts the total length of an Object[][] **totalNumberRatiosByWindowSize(int, int)** - Static method in class trans.misc.InefficientPseudoMedian: Precaluculates the total number of pairwise ratios for a range of window sizes. **totalObservations(HashMap<String, PointData>)** - Static method in class edu.utah.seq.data.PointData: Counts the total number of observations. **totalObservations(HashMap<String, PointData>[])** - Static method in class edu.utah.seq.data.PointData: Counts the total number of observations. **totalObservations2(HashMap<String, PointData[]>)** - Static method in class edu.utah.seq.data.PointData: Counts the total number of observations. **TPMapDensityReducer** - Class in trans.tpmap: Randomly drops a certain fraction of tpmap lines. **TPMapDensityReducer(String[])** - Constructor for class trans.tpmap.TPMapDensityReducer: **TPMapDuplicateFilter** - Class in trans.tpmap: For extracting duplicate oligo/ features from Affy's bpmap file, can be used as a stand alone but better to use `TPMapProcessor`. **TPMapDuplicateFilter(String)** - Constructor for class trans.tpmap.TPMapDuplicateFilter: For running this alone. **TPMapDuplicateFilter(File, File, String)** - Constructor for class trans.tpmap.TPMapDuplicateFilter: For running in combination with TPMapProcessor **TPMapFeature** - Class in trans.tpmap: Container for info about a particular oligo feature from a tpmap file. **TPMapFeature(String[])** - Constructor for class trans.tpmap.TPMapFeature: **TPMapLine** - Class in trans.tpmap: Minimal tpmap line, chrom, start, nothing else. **TPMapLine(String[])** - Constructor for class trans.tpmap.TPMapLine: For parsing from an Agilent data file. **TPMapLine(String, String, String, String)** - Constructor for class trans.tpmap.TPMapLine: For parsing from an Agilent promoter array data file. **TPMapLine(String, int, int, int, String)** - Constructor for class trans.tpmap.TPMapLine: Coordinate are zero based! **TPMapLine(String, int, int, int, String, boolean)** - Constructor for class trans.tpmap.TPMapLine: Coordinate are zero based! **TPMapLine(String[], boolean)** - Constructor for class trans.tpmap.TPMapLine: For parsing from an Nimblegen NDF data file. **TPMapLine(String)** - Constructor for class trans.tpmap.TPMapLine: **TPMapOligoBlastFilter** - Class in trans.tpmap: Application for remapping and filtering a text version bpmap file with BLAST. **TPMapOligoBlastFilter(String[])** - Constructor for class trans.tpmap.TPMapOligoBlastFilter: **TPMapProcessor** - Class in trans.tpmap: App for processing the tpmap file for use by downstream TiMAT applications. **TPMapProcessor(String[])** - Constructor for class trans.tpmap.TPMapProcessor: **TPMapSort** - Class in trans.tpmap: This sorts a tpmap first by chromosome, second by base position. **TPMapSort(String[])** - Constructor for class trans.tpmap.TPMapSort: **TPMapStripHighLowGC** - Class in trans.tpmap: Strips a tpmap file of lines where the %GC is > 0.6 or < 0.4. **TPMapStripHighLowGC()** - Constructor for class trans.tpmap.TPMapStripHighLowGC: **trans.anno** - package trans.anno: For associating intervals, binding regions, and binding peaks with GFF annotation. **trans.bkgrnd** - package trans.bkgrnd: For examining background GC content and ratio bias. **trans.cel** - package trans.cel: Apps and methods for manipulating cel files. **trans.graphics** - package trans.graphics: For graphically representing '.cel' files and intervals. **trans.main** - package trans.main: Principle classes and scripts used in TiMAT2 analysis. **trans.misc** - package trans.misc: Rough working scripts. **trans.qc** - package trans.qc: Quality control apps for cel files. **trans.roc** - package trans.roc: For manipulating the BAC dmel spike in data and generating ROC curves. **trans.tpmap** - package trans.tpmap: For maping, filtering, and calculating statistics about text version bpmap files. **Transcript** - Class in util.bio.annotation: Transcript relevant information, mRNA. **Transcript(int, int, String, int)** - Constructor for class util.bio.annotation.Transcript: **Transcript(Gff3Feature)** - Constructor for class util.bio.annotation.Transcript: **transform10Log10(double)** - Static method in class util.bio.calc.Alignment: Calculates a -10Log10(number) transformation **transformPMMM(float[])** - Static method in class trans.cel.QuantileNormalization: Performs a max(PM-MM,1) on the float array where MM is just after each PM. **TransformRatios** - Class in trans.misc: Transforms ratio scores in Intervals based on the BAC GC Corrector. **TransformRatios()** - Constructor for class trans.misc.TransformRatios: **TransGroup** - Class in util.bio.annotation: A TransGroup describes a product derived from a GeneGroup contains one transcript, possibly a translation, and a possibly a set of exons. **TransGroup(Transcript, Translation, ExonIntron[])** - Constructor for class util.bio.annotation.TransGroup: **TransGrpGlyph** - Class in gata.geneGlyphs: **TransGrpGlyph(TransGroup, GATAParams, AnnoSpecParams)** - Constructor for class gata.geneGlyphs.TransGrpGlyph: **Translation** - Class in util.bio.annotation: Translation specific information, protein but referenced with genomic coordinates thus some bits code for protein, some don't physically exist. **Translation(int, int, int)** - Constructor for class util.bio.annotation.Translation: **Translation(Gff3Feature)** - Constructor for class util.bio.annotation.Translation: **trim(String[])** - Static method in class util.gen.Misc: Trims all the strings in the array String.trim() **trimCommon(String[])** - Static method in class util.gen.Misc: Trims the comon chars from the front and back of each line, won't entirely delete line. **trimCommonStart(String[])** - Static method in class util.gen.Misc: Trims characters common to all from start of lines. **trimmedMean(double[], double)** - Static method in class util.gen.Num: Trims the fraction off the top and bottom, returns the mean of the remainder, rounds trimming # up. **trimmedMean(float[], double)** - Static method in class util.gen.Num: Trims the fraction off the top and bottom, returns the mean of the remainder, rounds trimming # up. **trimmedMean(double[], int)** - Static method in class util.gen.Num: Makes a trimmed mean, note the trim number is not a % but the number of values to drop from the beginning and end of the ordered set. **trimmedMean(float[], int)** - Static method in class util.gen.Num: Makes a trimmed mean, note the trim number is not a % but the number of values to drop from the beginning and end of the ordered set. **trimOutliers(float[], float, float)** - Static method in class util.gen.Num: Sets values exceeding the cutoff to the default. **trimOutliers(float[][], float, float)** - Static method in class util.gen.Num: For each f[replica][]Sets values exceeding the cutoff to the default. **trimQuotes(String)** - Static method in class util.gen.Misc: Trims "" from the beginning and end of String if both are found. **trimTxt(String)** - Static method in class util.gen.Misc: Removes .txt from end of String if present **truncate(String, int)** - Static method in class util.gen.Misc: Truncates a String adding three periods if too long. **tukeyBiWeight(double[])** - Static method in class util.gen.Num: Calculates a one-step tukey biweight estimator using 5 as the tuning constant and adding 0.0001 to the denominator to avoid dividing by zero, Affy's params.

---


|  |  |  |
| --- | --- | --- |
| **Overview** \| Package \| Class \| Use \| **Tree** \| **Deprecated** \| **Index** \| **Help** | |  |
| **PREV LETTER**   **NEXT LETTER** | **FRAMES**    **NO FRAMES**     **All Classes** |  |


A B C D E F G H I J L M N O P Q R S T U V W X Z 

---
